# Supplementary material for: Signaling and Mechanics influence the number and size of epithelial rosettes in the migrating zebrafish Posterior Lateral Line primordium
Source: bioRxiv. 2025 May 19:2025.05.17.650326. Preprint. [Version 1] doi: 10.1101/2025.05.17.650326 (PMC12190344; doi:10.1101/2025.05.17.650326)
Supplement: 1 [file NIHPP2025.05.17.650326V1-supplement-1.pdf]

bioRxiv preprint doi: https://doi.org/10.1101/2025.05.17.650326; this version posted May 20, 2025. The copyright holder for this preprint (which was not certified by peer review) is the author/funder. This article is a US Government work. It is not subject to copyright under 17 USC 105 and is also made available for use under a CC0 license.

Supplementary Figure Legends

Figure S1: (A) Panels showing rosettes fusing and splitting in an *sdf1a* morpholino-injected embryo. Membrane and nuclei are shown in green and magenta respectively. The constrictions of interest are marked by 1 (red) and 2 (blue). A stable depositing rosette is marked by an orange arrowhead, and a nascent protoneuromast that forms after constrictions 1 and 2 fuse is marked by a light blue arrowhead. **(B)** Tracking constrictions 1 and 2 over time shows them fusing and splitting up. Tracking starts when constriction 2 forms. Note that this leads to a slight shift in time in the panels **(A)** and **(B)** (Time = 0 in panel **(B)** corresponds to 126 mins. in panel **(A)**).

Figure S2: Evolution of turtle aggregation dynamics over time as a function of spring-constant of links.

Figure S3: Example simulation where lateral domain contractility ($\lambda_{fgflat-fgflat} = 40$) and cell-cell adhesion ($E_{fgflat-fgflat} = 1$) have been increased. Panels depict start (top), an interim timestep (middle), and end (bottom) of simulation. Relatively small rosettes are marked with arrows, and relatively larger ones are marked with brackets.

bioRxiv preprint doi: https://doi.org/10.1101/2025.05.17.650326; this version posted May 20, 2025. The copyright holder for this preprint (which was not certified by peer review) is the author/funder. This article is a US Government work. It is not subject to copyright under 17 USC 105 and is also made available for use under a CC0 license.

Appendix

Supplementary Tables

Table 1: Details of the agent-based model created in NetLogo

Variable Name	Physiological representation	Value
Wnter speed	Speed of leading cells	0, 0.015 – 0.018
FGFer speed	Speed of trailing cells	0, 0.015 – 0.018
PLLp width	PLLp width	5
PLLp length	PLLp length	30
Proliferation rate	Rate of cell divisions	5×10^{-4}
Wnt fraction	Fraction of PLLp with high Wnt activity	0.6 or 1
Wnt size		30
FGF size		30
Wnt spring constant	Adhesivity + Contractility of leading cells	0.14 – 0.2

bioRxiv preprint doi: https://doi.org/10.1101/2025.05.17.650326; this version posted May 20, 2025. The copyright holder for this preprint (which was not certified by peer review) is the author/funder. This article is a US Government work. It is not subject to copyright under 17 USC 105 and is also made available for use under a CC0 license.

FGF spring constant	Adhesivity + Contractility of trailing cells	0.14 – 0.2
Wnt spring length	Refers to the region of influence of intercellular adhesion in the leading and trailing domains	0.1
FGF spring length		0.1
Wnt repulsion constant	Refers to how close cells are allowed to come before being repelled	0.03
FGF repulsion constant		0.03
Wnt radius		1.25
FGF radius		1.25
Cluster threshold	For visualization purposes only	32
Density scale	For visualization purposes only	36

Table 2: Cell shape constraints of the CPM created in CompuCell3D

Parameter	Cell type	Strength (λ)	Target Value
Surface Area	Sheath	0.2	200
	Wnt	2	800
	Fgfapi	2	100
	Fgflat	0.1	400
	Fgfbas	2.5	100
	ECM	2	200
	Skin	2	400
	Muscle	20	400
Perimeter	Sheath	0.2	100
	Wnt	0.3	120
	Fgfapi	0.3	25
	Fgflat	0.5	100

bioRxiv preprint doi: https://doi.org/10.1101/2025.05.17.650326; this version posted May 20, 2025. The copyright holder for this preprint (which was not certified by peer review) is the author/funder. This article is a US Government work. It is not subject to copyright under 17 USC 105 and is also made available for use under a CC0 license.

	Fgfbas	0.2	25
	ECM	0.8	80
	Skin	1	100
	Muscle	1	80

Table 3: Cell-cell adhesion energies within the CPM

Parameter	Cell-Cell interaction	Contact Energy
Contact Energies	Wnt-Sheath	6
	Wnt-Wnt	1
	Wnt-Fgfapi	3
	Wnt-Fgflat	0.5
	Wnt-Fgfbas	3
	Wnt-ECM	5
	Wnt-Skin	5
	Wnt-Muscle	10
	Fgfapi-Sheath	6
	Fgfapi-Fgfapi	1
	Fgfapi-Fgflat	10
	Fgfapi-Fgfbas	20
	Fgfapi-ECM	20
	Fgfapi-Skin	20
	Fgfapi-Muscle	20
	Fgflat-Sheath	10
	Fgflat-Fgflat	[1, 5]
	Fgflat-Fgfbas	10
	Fgflat-ECM	20
	Fgflat-Skin	20
	Fgflat-Muscle	20
	Fgfbas-Sheath	5

bioRxiv preprint doi: https://doi.org/10.1101/2025.05.17.650326; this version posted May 20, 2025. The copyright holder for this preprint (which was not certified by peer review) is the author/funder. This article is a US Government work. It is not subject to copyright under 17 USC 105 and is also made available for use under a CC0 license.

	Fgfbas-Fgfbas	8
	Fgfbas-ECM	2
	Fgfbas-Skin	20
	Fgfbas-Muscle	10
	ECM-Sheath	8
	ECM-ECM	0.5
	ECM-Skin	6
	ECM-Muscle	1
	Sheath-Sheath	6
	Sheath-Skin	4
	Sheath-Muscle	20
	Skin-Skin	0
	Skin-Muscle	10
	Muscle-Muscle	1
Internal Contact Energies (only for compartmental FGF cells)	Fgflat-Fgfbas	1
	Fgfapi-Fgflat	1
	Fgfapi-Fgfbas	10

Table 4: Cell Migration details in CPM

Cell type	Migratory Force (λ_x) (+x-direction)	Cell fluctuation amplitude
Wnt	[-0.1, -2]	[10, 50]
Fgfbas	-3	30
Sheath	-1	50

Table 5: *FocalPointPlasticity* links details in CPM

Cell-cell interaction	Strength (λ)	Target distance	Maximum distance

bioRxiv preprint doi: https://doi.org/10.1101/2025.05.17.650326; this version posted May 20, 2025. The copyright holder for this preprint (which was not certified by peer review) is the author/funder. This article is a US Government work. It is not subject to copyright under 17 USC 105 and is also made available for use under a CC0 license.

Wnt-Wnt	5	10	200
Wnt-Fgflat	10	30	40
Fgfapi-Fgfapi	5	8	20
Fgflat-Fgflat	[10, 40]	8	100
Skin-Skin	10	15	100
ECM-ECM	4	10	40
Fgfapi-Fgflat	8	15	30
Fgflat-Fgfbas	8	15	30

Supplementary Movies

Movie S1:

Movie of a migrating wildtype control PLLp showing cell membranes (top), nuclei (middle), and merged (bottom) images.

Movie S2:

Movie of a migrating PLLp in a heat-shocked *Tg(hsp:sdf1a)* embryo showing cell membranes (top), nuclei (middle), and merged (bottom) images.

Movie S3:

Movie of the migrating wildtype control PLLp from Movie S1, showing cell membranes (top) and the corresponding PIV colormap overlaid on cell nuclei (bottom).

Movie S4:

bioRxiv preprint doi: https://doi.org/10.1101/2025.05.17.650326; this version posted May 20, 2025. The copyright holder for this preprint (which was not certified by peer review) is the author/funder. This article is a US Government work. It is not subject to copyright under 17 USC 105 and is also made available for use under a CC0 license.

Movie of a migrating PLLp in the heat-shocked *Tg(hsp:sdf1a)* embryo from Movie S2, showing cell membranes (top) and the corresponding PIV colormap overlaid on cell nuclei (bottom).

Movie S5:
Movie of a migrating PLLp in a standard control morpholino-injected embryo showing cell membranes (top), nuclei (middle), and merged (bottom) images.

Movie S6:
Movie of a migrating PLLp in a 2ng sdf1a morpholino-injected embryo showing cell membranes (top), nuclei (middle), and merged (bottom) images.

Movie S7:
Movie of a migrating PLLp treated with 20μM PP1 showing cell membranes (top) and the corresponding PIV colormap overlaid on cell nuclei (bottom).

Movie S8:
Movie of a representative agent-based model simulation. Simulation showing aggregation of turtles without migration (Top), with **WNTers** moving faster than **FGFers** (middle), and with **WNTers** moving slower than **FGFers** (bottom).

Movie S9:
Movie of a representative agent-based model simulation showing the shrinkage of **WNTers** and deposition of **Depositers**

Movie S10:
Movie of a representative Cellular Potts model PLLp simulating WT control conditions.

Movie S11:

bioRxiv preprint doi: https://doi.org/10.1101/2025.05.17.650326; this version posted May 20, 2025. The copyright holder for this preprint (which was not certified by peer review) is the author/funder. This article is a US Government work. It is not subject to copyright under 17 USC 105 and is also made available for use under a CC0 license.

Movie of a representative Cellular Potts model PLLp where leading cells slow down before resuming migration, thereby simulating the heat-shocked *Tg(hsp:sdf1a)* phenotype.

Movie S12:

Movie of a representative Cellular Potts model PLLp where an increase in lateral domain contractility and adhesion leads to formation of a large rosette.

bioRxiv preprint doi: https://doi.org/10.1101/2025.05.17.650326; this version posted May 20, 2025. The copyright holder for this preprint (which was not certified by peer review) is the author/funder. This article is a US Government work. It is not subject to copyright under 17 USC 105 and is also made available for use under a CC0 license.

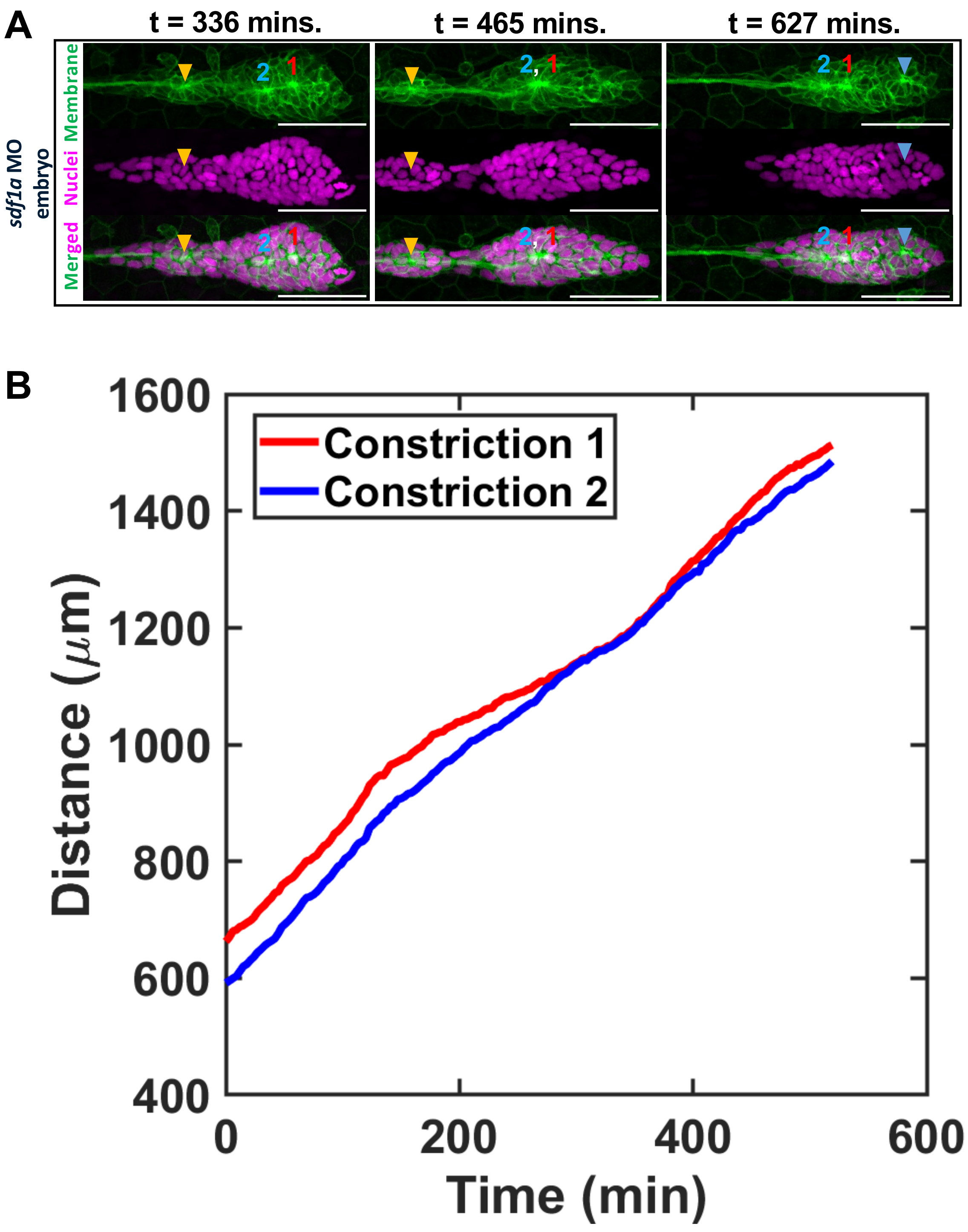

Supp Fig S1

bioRxiv preprint doi: https://doi.org/10.1101/2025.05.17.650326; this version posted May 20, 2025. The copyright holder for this preprint (which was not certified by peer review) is the author/funder. This article is a US Government work. It is not subject to copyright under 17 USC 105 and is also made available for use under a CC0 license.

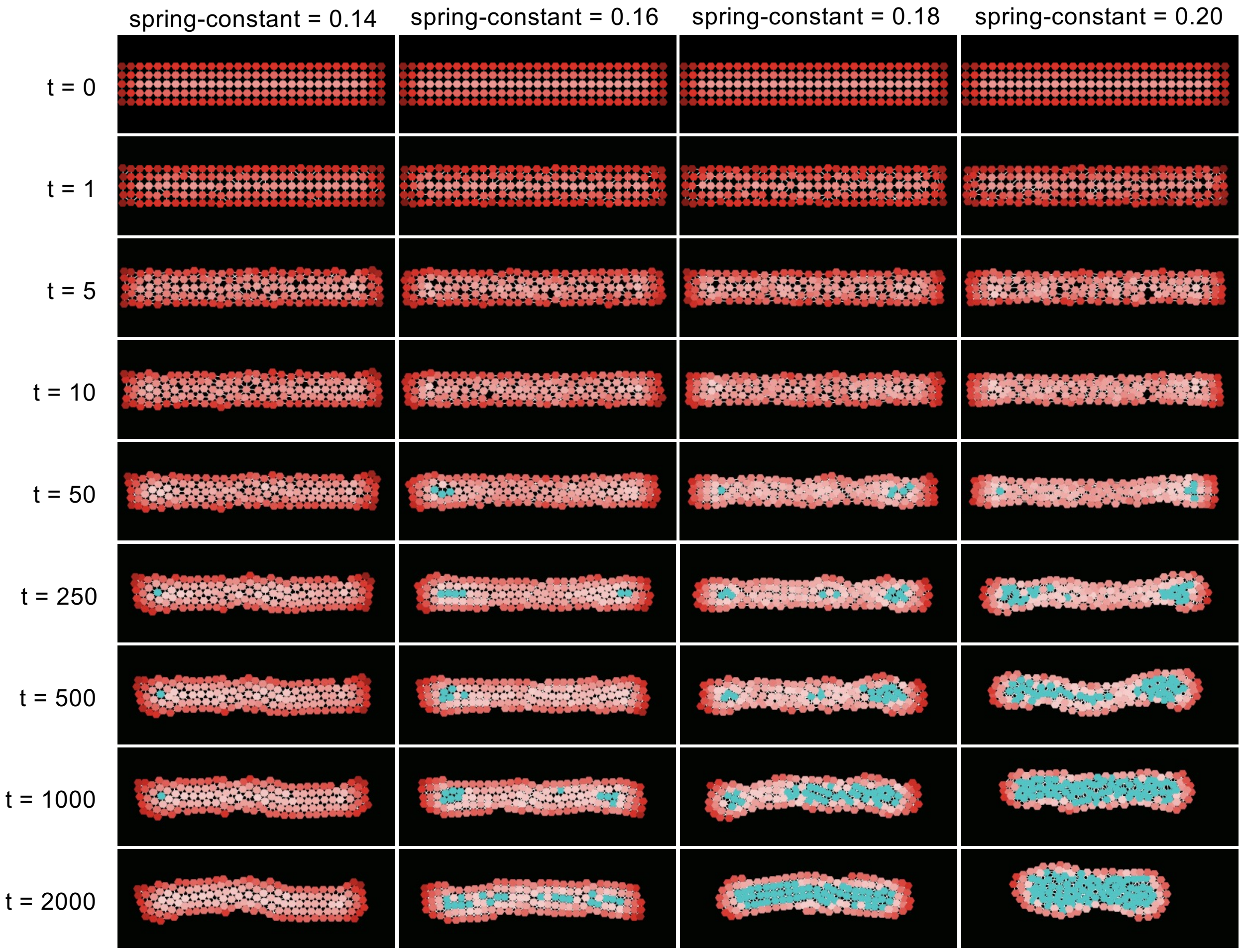

Supp Fig S2

bioRxiv preprint doi: https://doi.org/10.1101/2025.05.17.650326; this version posted May 20, 2025. The copyright holder for this preprint (which was not certified by peer review) is the author/funder. This article is a US Government work. It is not subject to copyright under 17 USC 105 and is also made available for use under a CC0 license.

Increase in lateral domain contractility and adhesion

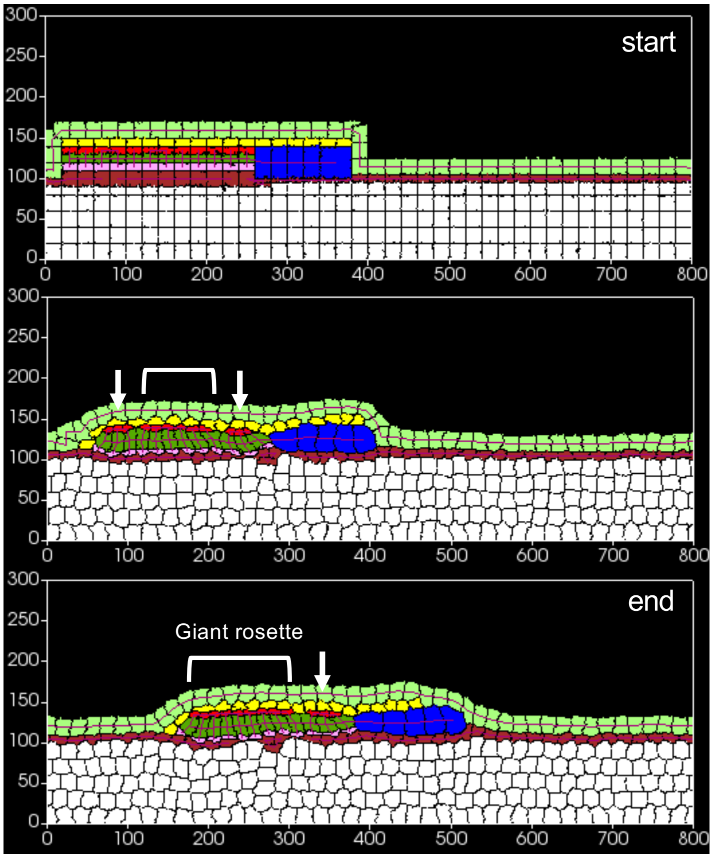

Supp Fig S3
